# Central relaxin-3/RXFP3 signaling potently drives orexigenic behaviors and attenuates cisplatin-induced anorexia and body weight loss

**DOI:** 10.1016/j.isci.2026.117785

**Published:** 2026-10-08

**Authors:** Keshav S. Subramanian, Drew Belser, Oleksandr Orativskyi, Ashley Lee, Genevieve Curtis, Schuylar Kamicha, Shion Yuki, Dennis Vu, Tsz Hang Ko, Bart C. De Jonghe, Robert P. Doyle, Caroline E. Geisler, Matthew R. Hayes

**Affiliations:** 1 University of Pennsylvania, Philadelphia, PA, USA; 2 Syracuse University, Syracuse, NY, USA; 3 University of Kentucky, Lexington, KY, USA; 4 State University of New York, Syracuse, NY, USA

**Keywords:** relaxin-3, rxfp3, neuropeptide, feeding, metabolism, meal patterns, food motivated behaviors, cisplatin

## Abstract

The relaxin-3 neuropeptide system promotes energy balance by increasing feeding behavior. Here, we investigate the behavioral mechanisms mediating the orexigenic effects of relaxin-3 and the therapeutic potential of relaxin-3 to combat metabolic disorders. We show that central and peripheral administration of relaxin-3 increases high-fat diet intake by increasing meal size in obese rats. Furthermore, we deleted relaxin family peptide receptor 3 (RXFP3) neuronally using RXFP3 fl/fl hSyn-Cre+ mice and show that neuronal RXFP3 is required for the orexigenic effects of relaxin-3. In addition, central relaxin-3 administration augmented food motivation for sucrose and hyperphagia in a binge-like eating model. We then evaluated the potential of relaxin-3 to drive food intake in rats receiving acute maladaptive chemotherapy. Pretreatment of central relaxin-3 attenuated cisplatin chemotherapy-induced anorexia and body weight loss. These findings characterize the behavioral mechanisms by which the relaxin-3 system drives feeding behaviors and highlight its therapeutic potential for metabolic wasting disorders.

## Highlights

• Relaxin-3 increases high-fat food intake by promoting larger meals
• Neuronal RXFP3 is required for relaxin-3-induced feeding
• Relaxin-3 enhances sucrose motivation and hyperphagia in a binge-like eating model
• Central relaxin-3 reduces chemotherapy-induced anorexia and body weight loss

## Graphical abstract

**Figure:**
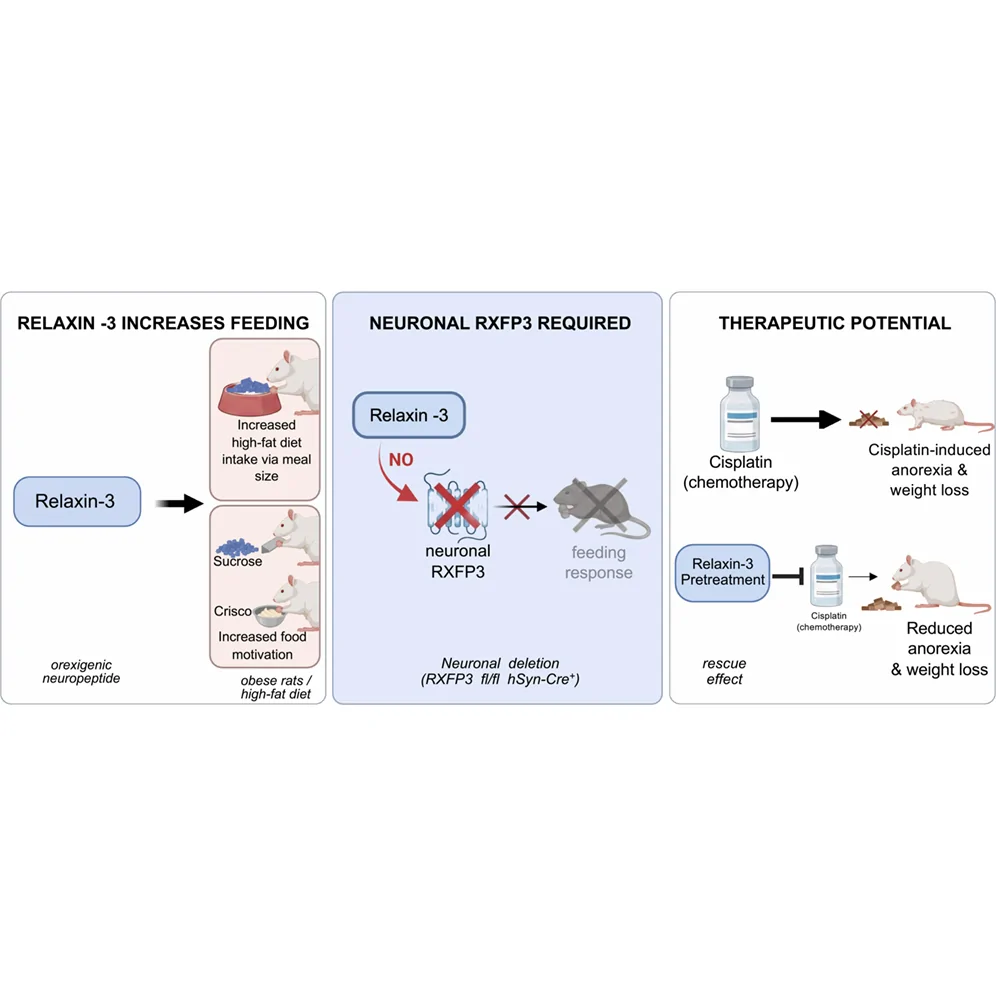

## Teaser

Molecular interaction; Molecular network; Molecular neuroscience

## Introduction

Relaxin-3 is an understudied neuropeptidergic system regulating a variety of behavioral and neuronal functions across energy balance and feeding behavior domains.^1^ Relaxin-3 is primarily produced centrally, with the largest population of relaxin-3-producing neurons being in the brainstem nucleus incertus (NI). Relaxin-3 expression is found in peripheral tissues, but it is at very low levels.^2^ The native G protein-coupled receptor (GPCR) for relaxin-3 is the relaxin family peptide receptor 3 (RXFP3); however, it can also bind to Rxfp1 and Rxfp4 at a lower affinity.^3^ RXFP3 is widely expressed throughout the brain and expressed in specific peripheral organs, most notably the gastrointestinal (GI) tract and endocrine tissue.^4^ In the brain, RXFP3 is expressed with the highest densities in feeding-relevant hypothalamic areas such as the paraventricular nucleus (PVN) of the hypothalamus, lateral hypothalamus (LHA), and arcuate nucleus (ARC).^3^^,^^5^ The hypothalamus is involved in regulating feeding and food-motivated behaviors, and relaxin-3 has been shown to be associated with these functions.^1^^,^^6^ Acute and chronic lateral intracerebroventricular (ICV) infusions of relaxin-3 pharmacologically stimulate increases in food intake and body weight gain in rats.^7^^,^^8^^,^^9^^,^^10^^,^^11^^,^^12^^,^^13^ In addition, intra-PVN administration of relaxin-3 also increases food intake.^12^^,^^14^Relaxin-3 mRNA levels in the NI are increased in obese rats^10^ and during stress-induced binge eating,^15^^,^^16^ while, conversely, RXFP3 antagonism prevents binge-like eating behavior in rats^15^^,^^16^ and reduces appetitive behaviors before a meal and chow consumption during mild food restriction in mice.^6^ Lastly, global RXFP3 knockout mice showed reduced operant responding for sucrose in a progressive-ratio (PR) task.^17^ Importantly, the underlying behavioral and physiological mechanisms that mediate the orexigenic effects of relaxin-3 remain unclear.

Here, we aim to characterize the mechanisms by which relaxin-3 promotes food intake and food-motivated behaviors. We first characterize the mechanisms by which relaxin-3 promotes high-fat diet (HFD) intake by examining meal patterns following both central and peripheral relaxin-3 administration. Furthermore, we generated RXFP3 fl/fl hSyn-Cre+ mice, which have the Rxfp3 gene deleted from all neuronal cells, to test the requirement of specifically neuronal RXFP3 for the orexigenic effects of relaxin-3. Additionally, using a PR task and binge-like eating paradigm, we evaluated the ability of relaxin-3 to promote food-motivated behaviors. Lastly, given the potent orexigenic effects of relaxin-3, we evaluated its therapeutic potential to combat metabolic wasting disorders that suppress food intake and body weight, such as cancer cachexia.^18^ While there has been some progress made in treating cancer cachexia by inhibiting the growth differentiation factor (GDF-15),^19^ there are no approved treatments for cancer cachexia, and there is a need to identify alternative therapeutic targets. Therefore, we evaluated the effects of relaxin-3 to attenuate the anorexigenic and food-intake-reducing effects of the anti-cancer chemotherapy cisplatin. Overall, this work highlights the neurobiological mechanisms by which the relaxin-3 system promotes food intake and food-motivated behaviors and the therapeutic potential of relaxin-3 to combat cancer-cachexia.

## Results

### Central relaxin-3 increases HFD intake via meal size in obese rats

We evaluated the effects of acute lateral ventricle administration of relaxin-3 on home cage HFD intake and meal patterns to determine which feeding microstructures are responsible for relaxin-3-induced hyperphagia. We evaluated two different doses of relaxin-3 (18 and 180 pmol) in lateral ventricle-cannulated male rats maintained on an HFD (Figure 1A). The high dose of relaxin-3 (180 pmol) augmented food intake every 30 min from 2 h up to 4 h post-injection and increased 24-h body weight in obese male rats (Figures 1B and 1C). The low dose of relaxin-3 (18 pmol) did not impact food intake at any time point or body weight (Figures 1B and 1C). Relaxin-3 (180 pmol) drove an increase in average meal size every 30 min up to 3 h post-injection (Figure 1D) and an increase in average meal duration at 1, 4, and 5 h post-injection (Figure 1E). Importantly, relaxin-3 (180 pmol) did not impact meal frequency at any time point (Figure 1F). This suggests that relaxin-3 (180 pmol) promoted consumption within the context of a meal not between meals. Furthermore, since relaxin-3 (180 pmol) drove food intake at early time points, we evaluated the effect of relaxin-3 (180 pmol) on the first eating events in the dark cycle. Relaxin-3 (180 pmol) significantly increased the size of the first meal (Figure 1G) by potentially increasing first meal duration (Figure 1H), though that did not reach statistical significance. Furthermore, there was no difference in the rate of intake during the first meal (Figure 1I). These data support that the orexigenic response of central relaxin-3 (180 pmol) is through augmenting meal size and duration at early time points in obese male rats.

**Figure 1:**
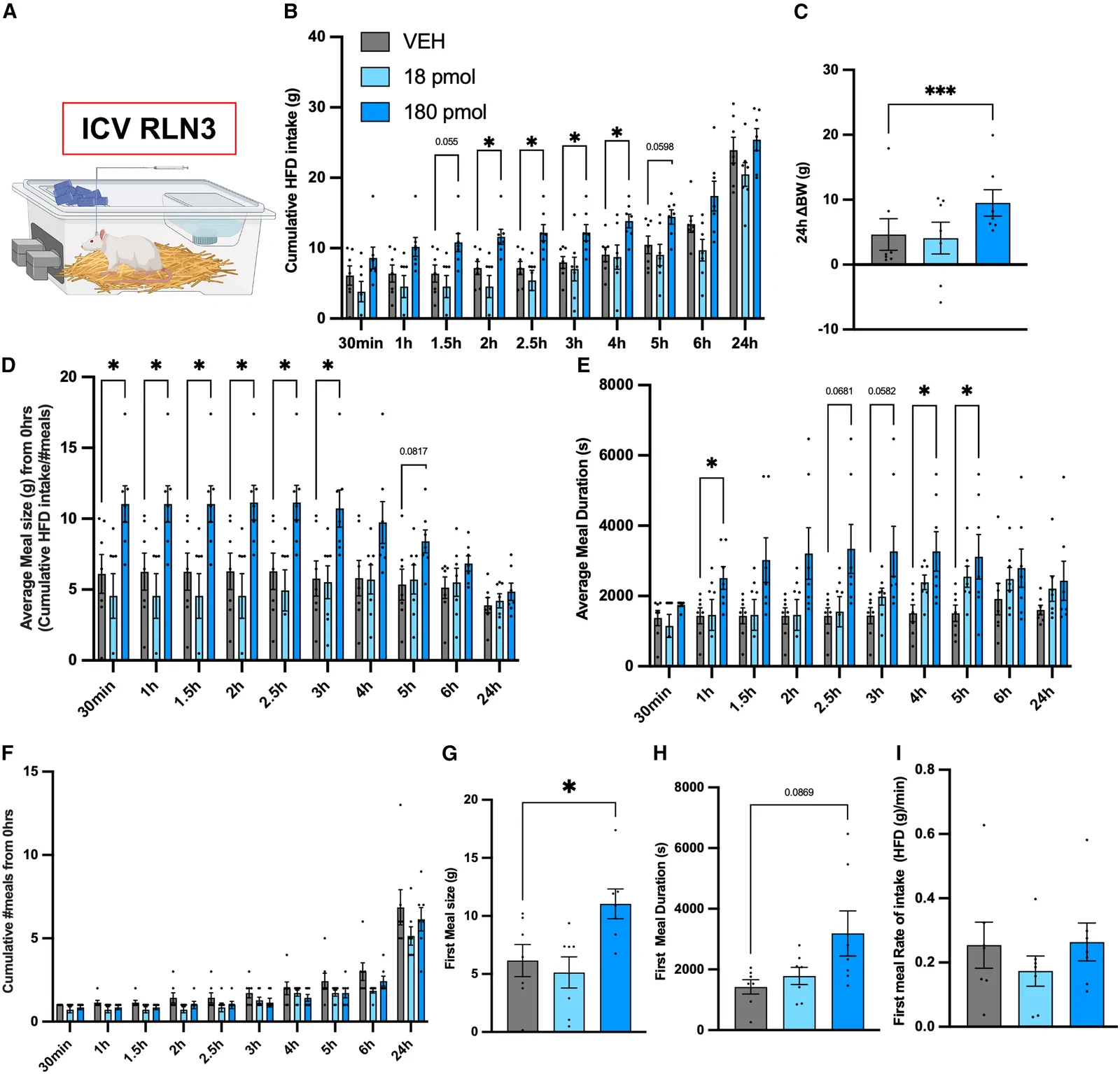
Central relaxin-3 potently increases high-fat diet intake via meal size in obese rats (A) Schematic diagram depicting ICV lateral administration of relaxin-3 in obese rats maintained on a high-fat diet (HFD) and measuring food intake and meal patterns (*n* = 7). (B) Cumulative HFD intake, (C) 24-h body weight change, (D) average meal size, (E) meal duration, and (F) meal frequency from 0 to 24 h post-injection. (G–I) Size (G), duration (H), and rate of intake (I) of the first meal. Dose response of lateral ventricle-administered relaxin-3 (18 pmol and 180 pmol/2 μL) in obese rats maintained on an HFD. Data are mean ± SEM. Each experiment was within subjects; *n* = 7 per treatment. Data were analyzed by one-way (C, G, and H) or two-way (B and D–F) ANOVA with Dunnett’s or SNK post hoc test. ∗*p* ≤ 0.05, ∗∗∗*p* ≤ 0.001. Veh, vehicle; h, hour.

### Systemic relaxin-3 augments HFD intake via meal size in obese rats

We then assessed the effects of systemic subcutaneous (SC) administration of relaxin-3 on home cage HFD intake and meal patterns. We evaluated two different doses of relaxin-3 (180 and 1,800 pmol/kg) in male rats maintained on an HFD (Figure 2A). The low dose of relaxin-3 (180 pmol) potently augmented food intake at 3 h (Figure 2B). Relaxin-3 (180 pmol) did not impact 24-h body weight in obese rats (Figure 2C). Interestingly, the high dose of relaxin-3 (1,800 pmol) did not significantly impact food intake or body weight in obese male rats (Figures 2B and 2C). Notably, systemic relaxin-3 (180 pmol) did not augment food intake as as potently central relaxin-3, suggesting differences because of brain penetrance. Relaxin-3 (180 pmol) did not reach statistical significance in driving an increase in average meal size (Figure 2D) or average meal duration (Figures 2D and 2E). Furthermore, relaxin-3 (180 pmol) did not impact meal frequency (Figure 2F). Lastly, relaxin-3 (180 pmol) did not augment first meal size or first meal duration (Figures 2G and 2H). These data support that the orexigenic response to systemic relaxin-3 (180 pmol) is through potentially augmenting meal size and duration.

**Figure 2:**
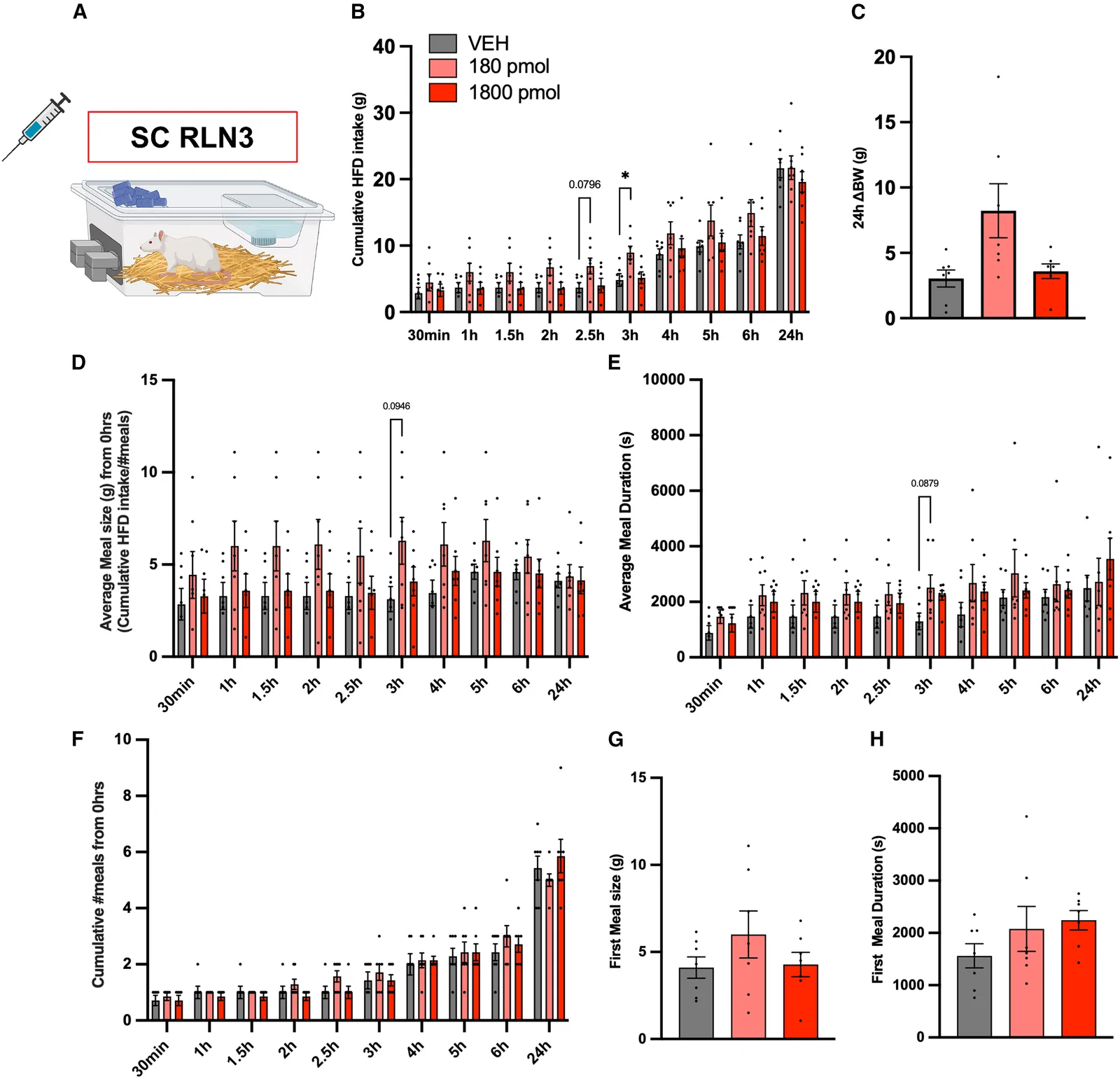
Systemic relaxin-3 augments high-fat diet intake via meal size in obese rats (A) Schematic diagram depicting subcutaneous administration of relaxin-3 in obese rats maintained on a high-fat diet (HFD) and measuring food intake and meal patterns (*n* = 7). (B–F) Cumulative HFD intake (B), 24-h body weight change (C), average meal size (D), meal duration (E), and meal frequency (F) from 0 to 24 h post-injection. (G and H) Size (G) and duration (H) of the first meal. Dose response of subcutaneously administered relaxin-3 (180 and 1,800 pmol/kg) in obese rats maintained on an HFD. Data are mean ± SEM. Each experiment was within subjects; *n* = 7 per treatment. Data were analyzed by one-way (C, G, and H) or two-way (B and D–F) ANOVA with Dunnett’s or SNK post hoc test. ∗*p* ≤ 0.05. Veh, vehicle; h, hour.

### Central RXFP3 is necessary for the orexigenic effects of relaxin-3

RXFP3 is highly conserved between mice, rats, and humans.^20^^,^^21^^,^^22^ We first evaluated the effects of acute systemic intraperiotenal (IP) administration of relaxin-3 in diet-induced obese (DIO) wild-type male mice. We tested two different doses of relaxin-3 (180 and 1,800 pmol/kg) in DIO male mice maintained on HFD. The low dose of relaxin-3 (180 pmol) potently drove food intake at 1, 3, and 6 h, and without significantly impacting 24-h body weight (Figures 3B and 3C). Similar to systemic relaxin-3 in obese male rats, the high dose of relaxin-3 (1,800 pmol) did not impact food intake or body weight in DIO male mice (Figures 3B and 3C). To confirm the role of neuronal RXFP3 signaling in mediating the orexigenic effects of relaxin-3, we generated neuronal-specific RXFP3 knockout mice (RXFP3 fl/fl hSyn-Cre+) by crossing Rxfp3 fl/fl mice^23^ and mice expressing Cre recombinase under the Synapsin promoter (hSyn) that drives recombinase in neurons. RXFP3 is primarily expressed neuronally^3^ but is also expressed in astrocytes^24^ and in the GI tract, both of which would remain intact in these mice.^4^^,^^25^ RT-qPCR confirmed ∼82%–83% reduction in RXFP3 mRNA in the hindbrain, hypothalamus, and ventral hippocampus compared to controls (Figures S1A and S1B). Both doses of relaxin-3 (180 and 1,800 pmol) had no impact on food intake (Figure 3E) and body weight (Figure 3F) in Rxfp3 fl/fl hSyn-Cre+ male mice. These data support the hypothesis that the neuronal RXFP3 is required to mediate the orexigenic effects of relaxin-3.

**Figure 3:**
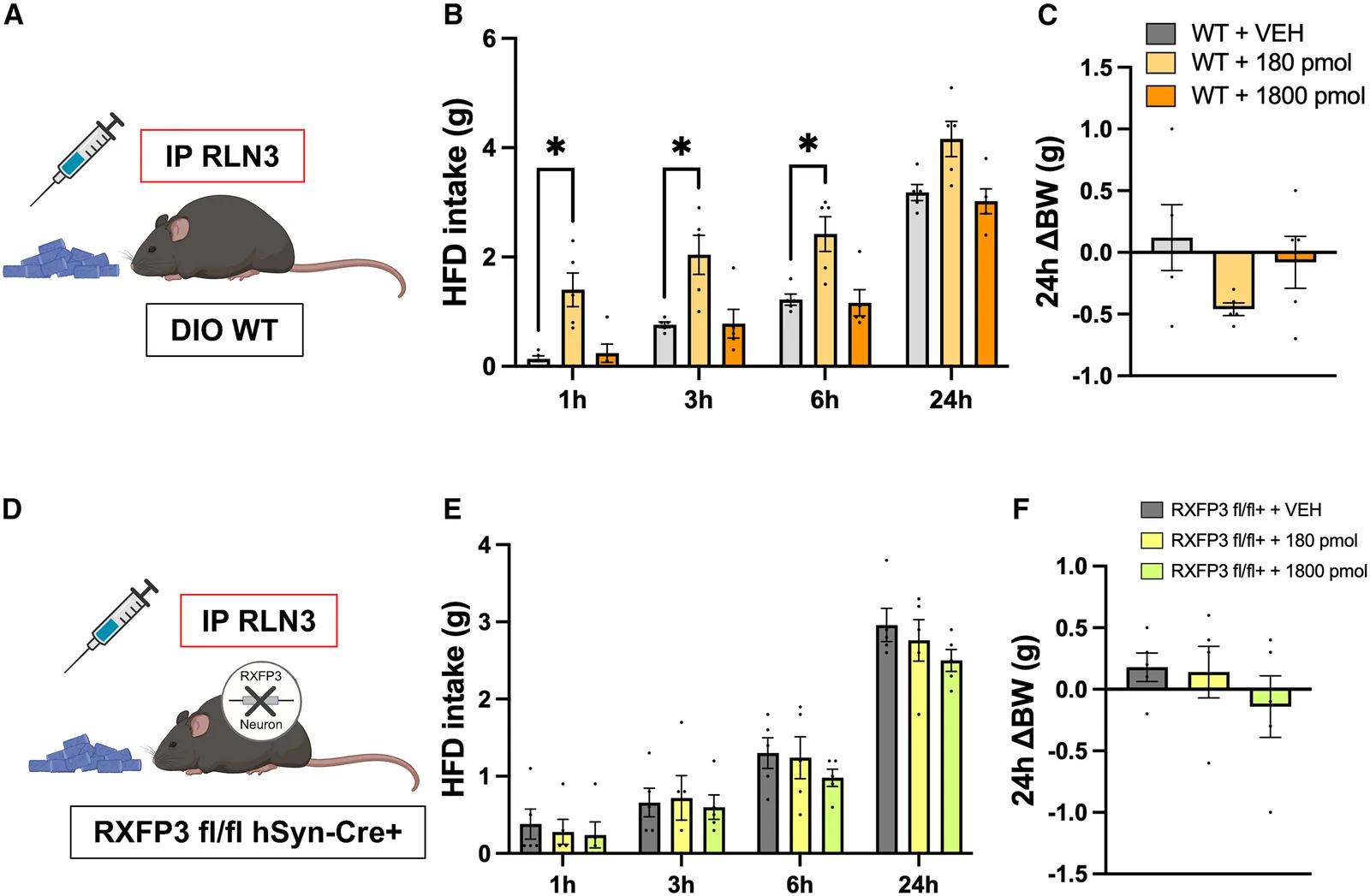
Central RXFP3 is necessary for the orexigenic effects of relaxin-3 (A) Schematic diagram depicting intraperitoneal administration of relaxin-3 in diet-induced obese (DIO) mice maintained on a high-fat diet (HFD) and measuring food intake (*n* = 5). (B and C) Cumulative HFD intake (B) and 24-h body weight change (C). (D) Schematic diagram depicting intraperitoneal administration of relaxin-3 in RXFP3 fl/fl hSyn-Cre+ mice maintained on an HFD and measuring food intake (*n* = 5). (E and F) Cumulative HFD intake (E) and 24-h body weight change (F). Dose response of intraperitoneally administered relaxin-3 (180 and 1,800 pmol/kg) in DIO and RXFP3 fl/fl hSyn-Cre+ mice maintained on an HFD. Data are mean ± SEM. Each experiment was within subjects; *n* = 5 per treatment. Data were analyzed by one-way (C and F) or two-way (B and E) ANOVA with Tukey or Dunnett’s post hoc test. ∗*p* ≤ 0.05, Veh, vehicle; 180 pmol and 1,800 pmol = relaxin-3 (180 pmol and 1,800 pmol)/100g. h, hour.

### Central relaxin-3 promotes food-motivation for palatable foods

The abovementioned findings showcase that relaxin-3 acts through Rxfp3 to potently drive HFD intake by augmenting meal size. We then wanted to test if relaxin-3 could influence other aspects of feeding behavior, including food-motivated behaviors. We tested lateral ventricle-cannulated male rats in the operant PR reinforcement schedule task, which is an established paradigm for measuring motivation to respond for a sucrose pellet^26^ (Figure 4A). We evaluated two different doses of relaxin-3 (18 and 180 pmol) centrally and showed that the high dose of relaxin-3 (180 pmol) increased the breakpoint (Figure 4B) and sucrose pellets earned (Figure 4C), without impacting incorrect lever presses (Figure 4D); importantly, the low dose of relaxin-3 (18 pmol) did not impact motivational responding in the PR task. This suggests that ICV relaxin-3 (180 pmol) increases motivation to work for palatable foods. To further test the effects of relaxin-3 signaling to drive food-motivated behaviors, we had lateral ventricle-cannulated male rats undergo a binge-eating paradigm whereby half the rats received daily access to the highly palatable, fatty food, Crisco (control), and the other half received every other day access to Crisco (binge) (Figure 4E). During the test day, relaxin-3 (360 pmol) augmented crisco intake, but not chow intake, within each group (Figures 4F and 4G); for both crisco and chow intake, there was a group effect where the binge group consumed more compared to the control group (Figures 4F and 4G). Furthermore, relaxin-3 (360 pmol) had no effect on 24-h body weight within each group, and there was no group effect on 24-h body weight (Figure 4H). These data overall suggest that central relaxin-3 drives food-motivated behaviors for highly palatable, calorically dense foods.

**Figure 4:**
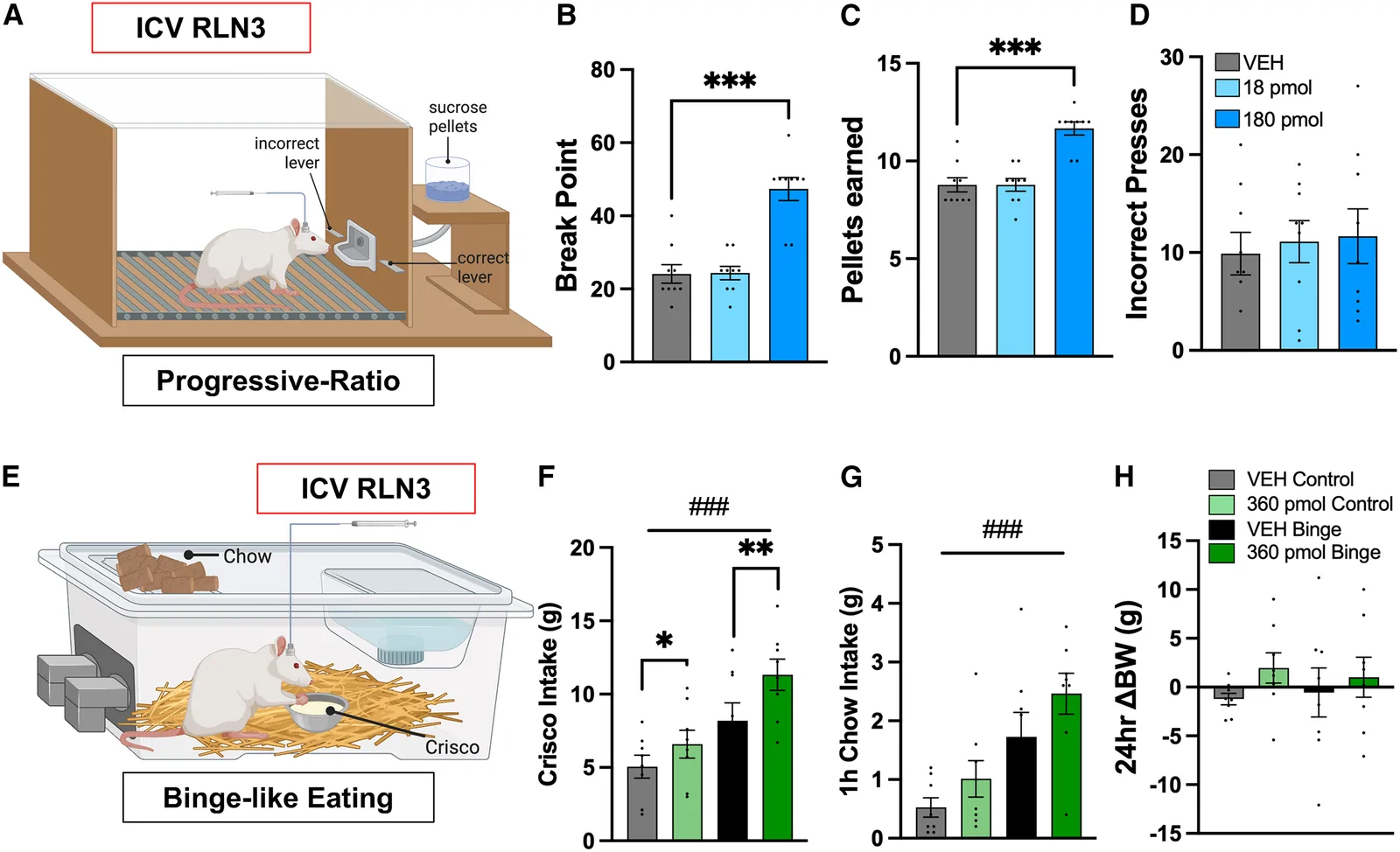
Central relaxin-3 promotes food-motivation for palatable foods (A) Schematic diagram depicting ICV lateral administration of relaxin-3 during a progressive-ratio task for sucrose (*n* = 9). (B–D) Breakpoint (B), pellets earned (C), and incorrect lever presses (D) during the progressive-ratio task. (E) Schematic diagram depicting ICV lateral administration of relaxin-3 during a binge-like eating paradigm (*n* = 8 per group). (F–H) Crisco intake (F), chow intake 0–1 h post-injection (G), and 24-h body weight change (H). Lateral ventricle administration of relaxin-3 (18, 180, and 360 pmol/2 μL) in rats during a progressive-ratio task and binge-eating paradigm. Data are mean ± SEM. Data were analyzed by one-way ANOVA (B–D) with Dunnett’s post hoc and unpaired and paired *t* tests (F–H). ∗*p* ≤ 0.05, ∗∗*p* ≤ 0.01, ∗∗∗*p* ≤ 0.001, and ### significant difference between binge and control groups (*p* ≤ 0.001). Veh, vehicle; 18 pmol, 180 pmol, and 360 pmol = relaxin-3 (18 pmol, 180 pmol, and 360 pmol/2 μL).

### Central relaxin-3 attenuates cisplatin-induced anorexia and body weight loss

Given that our findings strongly elucidate that relaxin-3 can drive food consumption, we were interested in understanding the therapeutic potential of relaxin-3 to promote a positive energy balance under disease conditions, which have anorexigenic effects. Patients with cancer are often very malnourished and suffer from cachexia (wasting syndrome). However, there are currently no FDA-approved drugs for the management of cancer cachexia. Due to the potent orexigenic effects of relaxin-3, we aimed to determine if relaxin-3 could counteract anti-cancer chemotherapy-induced anorexia and body weight loss. Male rats were implanted with cannulas in the lateral ventricle and were pretreated with ICV relaxin-3 (180 pmol) or vehicle (aCSF), followed by systemic (IP) acute treatment of cisplatin (6 mg/kg) or vehicle (0.9% saline) (Figure 5A). Central relaxin-3 by itself did not impact food intake nor body weight and did not stimulate kaolin intake relative to other controls (Figures 5B–5D). Furthermore, systemic cisplatin by itself potently reduced chow intake and body weight at all time points and augmented kaolin intake at 6 and 24 h relative to saline treatment animals (Figures 5B–5D), which aligns with previous cisplatin action in rat models.^27^ Post-hoc analyses revealed that central relaxin-3 pretreatment effectively combated cisplatin-induced anorexia, resulting in increased chow intake at 3, 6, and 24 h relative to cisplatin treatment animals. Furthermore, central relaxin-3 pretreatment did not attenuate kaolin intake at any time point but did reduce cisplatin-induced body weight loss at 24 h (Figure 5C). Central relaxin-3 did not attenuate the anorexigenic effects of either LiCl (Figures S2A–S2D) or GDF-15 administration (Figures S3A–S3D). Overall, these data suggest that central relaxin-3 pretreatment attenuated cisplatin-induced anorexia and body weight loss without impacting illness effects, highlighting the therapeutic potential of relaxin-3 to combat cancer cachexia.

**Figure 5:**
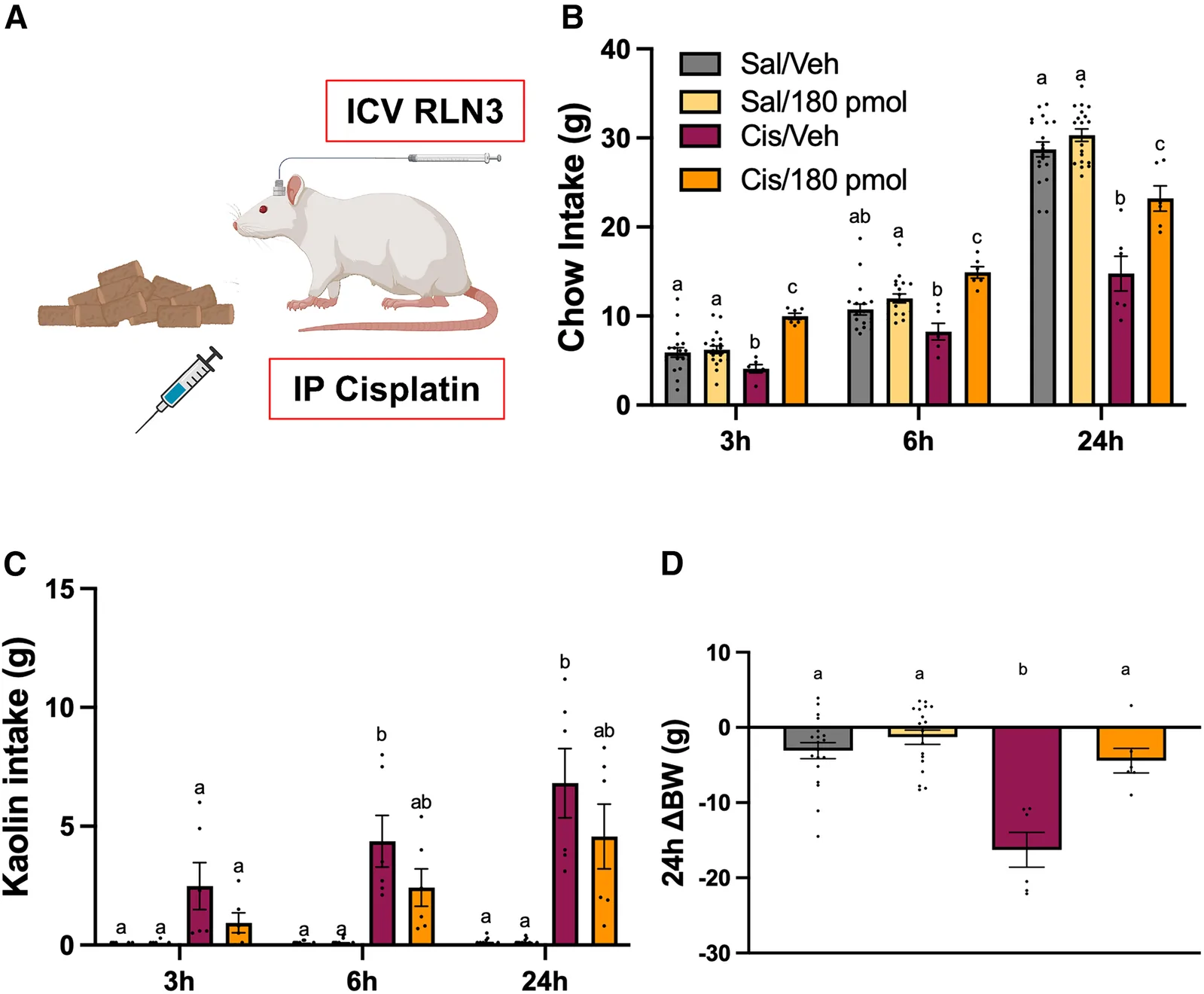
Central relaxin-3 attenuates cisplatin-induced anorexia and body weight loss (A) Schematic diagram depicting pretreatment with ICV lateral administration of relaxin-3 with systemic intraperitoneal administration of cisplatin in rats maintained on standard chow (*n* = 6–8 per group). (B–D) Chow intake (B), kaolin intake (C), and 24-h body weight change (D). Central relaxin-3 (180 pmol/2 μL) administration attenuates cisplatin (6 mg/kg)-induced anorexia and body weight loss. Data are mean ± SEM. Data were analyzed by two-way (B–D) ANOVA with Tukey’s post hoc. Bars that do not share a common letter (a, b, and c) within a time point differ significantly (*p* ≤ 0.05). Sal, saline; Veh, vehicle; Cis, cisplatin; 180 pmol = relaxin-3 (180 pmol/2 μL); h, hour.

## Discussion

The underlying mechanisms driving the orexigenic effects of relaxin-3 were investigated, and its therapeutic potential to drive food intake under metabolic-wasting conditions (chemotherapy-induced anorexia) was examined. The orexigenic effects of both central and peripheral administration of relaxin-3 were driven by an increase in meal size (trending for peripheral) and not in meal frequency, suggesting that relaxin-3 acts within a meal to increase consumption in obese rats. In addition, central relaxin-3 had a particularly potent effect on first meal parameters. Furthermore, using RXFP3 fl/fl hSyn-Cre+ mice, we show that deletion of neuronal RXFP3 blocks the orexigenic effects of relaxin-3 administration in obese mice. Relaxin-3 not only drives homeostatic feeding behaviors but also food-motivated behaviors. Following central relaxin-3 administration, rats show augmented motivation to work for sucrose in a PR task and hyperphagia for palatable foods in both a consistently offered and binge-like eating model. Lastly, pretreatment of central relaxin-3 attenuated the body weight-reducing and anorexigenic effects of acute administration of cisplatin, highlighting its ability to drive feeding under disease conditions that cause negative energy balance, such as cancer cachexia. Collectively, these results reveal the behavioral and physiological mechanisms by which relaxin-3 drives both home-cage feeding and food-motivated behaviors and showcases its potential to promote a positive energy balance under disease conditions that cause anorexia and body weight loss.

Cancer and its associated co-morbidities are one of the leading causes of death in the world,^28^ and among the most common co-morbidities is cachexia, with it impacting up to 80% of all patients with late-stage cancer^29^ and contributing to over 20% of cancer-related deaths.^30^ Cancer cachexia is a secondary disease driven not only by malignant tumors but also commonly by chemotherapy used to treat cancer, which collectively causes malnourishment, anorexia, and body weight loss.^30^ Together, these unwanted symptoms are associated with an overall poor prognosis, as they not only weaken the effects of anti-cancer chemotherapy but also increase its toxic side effects, leading to increased mortality in patients with cancer.^30^ Pomsegromab, a humanized antibody inhibiting GDF-15, has shown promise in combating cancer cachexia through improving energy balance and activity.^19^ However, it is currently unapproved, and there is a strong need to identify alternative therapeutic targets. In these studies, we show that relaxin-3 can drive food intake and food-motivated behaviors and, therefore, reasoned that relaxin-3 would be able to drive feeding while in an anorexigenic and body weight-reducing disease state driven by chemotherapy. Indeed, our results reveal that pre-treatment of relaxin-3 can attenuate the anorexigenic and body weight-reducing effects of acute cisplatin treatment. Importantly, relaxin-3 by itself did not cause an increase in food intake; however, in this study, we injected saline at 1mg/2 mL and dosed at 6 mg/kg, resulting in a large volume of saline being injected per animal prior to relaxin-3 treatment. This could be blunting the orexigenic effects of relaxin-3 because of the osmolarity and salt content of the saline, as relaxin-3 has been shown to be a potent mediator of salt appetite.^31^ Overall, this study highlights the potency of the orexigenic effects of relaxin-3 to be able to drive food intake even under maladaptive conditions and, importantly, did not further drive pica (kaolin intake) behavior.

Furthermore, the studies shown here highlight the potent orexigenic effects of relaxin-3 and its ability to drive homeostatic feeding and food-motivated behaviors. While relaxin-3 can drive feeding behaviors to combat chemotherapy-induced anorexia and body weight loss, an alternative therapeutic avenue is to antagonize relaxin-3 as a therapy for obesity and type 2 diabetes mellitus (T2DM). Some studies have shown that RXFP3 antagonism blocks the orexigenic effects of relaxin-3^11^^,^^32^^,^^33^ and can reduce food consumption and food-motivated behaviors.^6^^,^^15^ Given the increasing prevalence of obesity and T2DM in the US,^34^^,^^35^ future studies are warranted to investigate relaxin-3 antagonism as a potential therapeutic for obesity and T2DM.

Relaxin-3 primarily acts through its native GPCR, RXFP3, to exert its effects on energy balance.^3^ While RXFP3 is primarily thought of being expressed in the brain, recent studies have suggested that it is also expressed in peripheral organs. In particular, it has been shown that RXFP3 expression is present in the GI tract,^25^ including the enteroendocrine cells.^4^ Our results reveal that neuronal RXFP3 expression is required for the orexigenic effects of peripheral administration of relaxin-3. This suggests that relaxin-3 acts through RXFP3 expressed in the brain to exert its orexigenic effects and, therefore, that RXFP3 expressed in the GI tract may not be necessary for the orexigenic effects of relaxin-3 and may serve an alternative metabolic function independent of feeding behavior. In corroboration of this, we see that central administration of relaxin-3 had much more potent effects on HFD intake than systemic peripheral administration of relaxin-3, which would have much less brain penetrance. Consequently, further investigation is needed to elucidate the role of peripheral RXFP3 on the energy balance effects of relaxin-3.

Our results reveal that systemic administration of relaxin-3 drives HFD intake in both obese rats and mice. Interestingly, in both models, the lower dose (180 pmol/kg) but not the higher dose (1,800 pmol/kg) of relaxin-3 was able to increase HFD intake. This could be due to higher doses of relaxin-3 having off-target effects. Relaxin-3 primarily binds to RXFP3, which is densely expressed in the brain.^3^ However, it can also bind to RXFP1 at a lower affinity.^36^^,^^37^ RXFP1 is densely expressed in peripheral organs^38^^,^^39^^,^^40^ and it has been shown that augmented RXFP1 signaling can have anorexigenic effects in rodents.^41^ Therefore, higher concentrations of systemic relaxin-3 administration might be enhancing RXFP1 signaling and negating the orexigenic effects of relaxin-3/RXFP3 signaling. An additional consideration is that very high concentrations of relaxin-3 may be causing desensitization of RXFP3, resulting in reduced effectiveness. This has been previously shown in the relaxin receptor family, as RXFP1-agonist-induced activation led to desensitization and internalization of RXFP1.^42^

Furthermore, the therapeutic translation of relaxin-3/RXFP3 signaling for the treatment of metabolic disorders is likely to depend on the development of synthetic RXFP3 ligands with improved drug-like properties relative to the relaxin-3 neuropeptide. Relaxin-3 is a neuropeptide that exhibits high affinity and potency at RXFP3 but is limited by lower metabolic stability, rapid proteolytic degradation, and limited blood-brain barrier (BBB) penetration.^43^ To this end, our results indicate that centrally or systemically administered relaxin-3 has its most potent effects on feeding and food-motivated behaviors within the first 6 h post-injection. Consequently, substantial medicinal chemistry efforts have focused on generating stabilized peptide agonists and antagonists that retain RXFP3 pharmacology while improving pharmacokinetic properties such as serum stability, extended half-life, and improved BBB penetration.^33^^,^^43^^,^^44^ Indeed, *in vivo,* RLX-33, a small-molecule RXFP3 antagonist, blocked RXFP3 agonist-induced feeding.^45^ These advances suggest that both stabilized peptide analogs and emerging small-molecule RXFP3 modulators may overcome key limitations of native relaxin-3 and provide more clinically feasible approaches for therapeutic manipulation of the relaxin-3/RXFP3 system in related metabolic disorders.

Furthermore, the orexigenic effects of RXFP3 signaling have also been shown to have species differences. Central administration of RXFP3 agonists potently augments food intake^12^^,^^46^ and can be blocked by RXFP3 antagonism in rats.^11^^,^^33^^,^^46^ Interestingly, while central administration of RXFP3 antagonists blocks food intake and food-motivated behaviors in mice, ICV and intra-PVN injection of RXFP3 agonists had no effect on food intake.^6^ Central RXFP3 distribution is highly conserved across mice and rat brains, including hypothalamic feeding centers,^5^ but the neurochemical identity of these RXFP3-positive neurons and their associated connections remains unclear and could be a reason for differing effects on feeding behavior.^1^ In addition, central RXFP1 expression varies across mice and rats^47^ and should be considered when evaluating the orexigenic effects of relaxin-3, which can act on both receptors.

Another important consideration is that relaxin-3 can drive arousal and motivated behaviors.^48^^,^^49^ ICV infusion of a RXFP3 agonist promoted locomotor activity, exploration, and wakefulness in rodents^50^^,^^51^ and deletion of the RXFP3 gene increased sleep activity and reduced running wheel activity.^52^ Furthermore, micro-stimulation of the NI, where relaxin-3-producing neurons are densely found, increased locomotor behavior.^53^ Collectively, this suggests that relaxin-3 can broadly promote arousal behaviors, and given the overlap of arousal and motivational systems on food intake, it warrants future studies and consideration when evaluating the orexigenic effects of relaxin-3.

These results highlight relaxin-3 as a neuropeptidergic system that regulates energy balance. Relaxin-3 is highly effective at promoting a positive energy balance by increasing food intake via meal size and food-motivated behaviors through neuronal RXFP3. The potent orexigenic effects of relaxin-3 might be advantageous for patients suffering from loss of body weight and appetite from diseases such as cancer cachexia and/or chemotherapy; overall improving their prognoses and quality of life. On the other hand, for patients who suffer from obesity and T2DM, relaxin-3 antagonism may prove to be beneficial for improving weight loss and reducing food intake. Thus, there is a need to fully understand the different or overlapping mechanisms by which relaxin-3 regulates food intake for a variety of metabolic disorders.

### Limitations of the study

Importantly, it has been shown that relaxin-3 can have orexigenic effects in both male and female rodents, and studies have shown that there are sex-specific effects. Indeed, ICV relaxin-3 administration more potently increased food intake and Body weight (BW) in female rats compared to male rats.^54^ Furthermore, a combination of restraint stress, repeated food restriction, and intermittent access to palatable food led to an increase in relaxin-3 (RLN3) mRNA in the NI and hyperphagia in female rats but not in male rats.^16^ Given that the relaxin-3 system has been shown to have sex-specific effects on feeding, future evaluation of potential sex differences in relaxin-3 mediation of food-motivated behaviors is warranted.

Furthermore, combination treatment of ICV relaxin-3 and high-volume intraperitoneal saline injection did not augment chow intake in the cisplatin (Figure 5B) and the LiCl study (Figures S2B). Notably, relaxin-3 has been shown to be a potent mediator of salt appetite^31^ and appears to have more potent effects with highly palatable, calorically dense foods compared to standard chow, though this warrants further investigation. Therefore, a combination of bodily osmolarity and food palatability/caloric-density preference may be impacting the orexigenic effects of central relaxin-3 and necessitates future studies.

In addition, ICV relaxin-3 and cisplatin were administered concurrently, and while the results are very noteworthy, a more realistic clinical application would be to administer relaxin-3 either prior to cisplatin administration or after the onset of anorexigenic symptoms induced by cisplatin to see if it either blocks or reverses the effects of cisplatin. Furthermore, future studies should also investigate relaxin-3 neuronal activity following cisplatin administration.

As noted in the discussion section, higher concentrations of relaxin-3 could be engaging RXFP1 signaling, in addition to RXFP3, leading to off-target feeding-relevant effects. Therefore, future studies should focus on selective RXFP3 activation using selective agonists and antagonists and compare their behavioral and molecular mechanisms to those of the native relaxin-3 peptide, including assaying for expression level in the brain.

## Resource availability

### Lead contact

Requests for further information and resources and reagents should be directed to and will be fulfilled by the lead contact, Matthew R. Hayes (hayesmr@pennmedicine.upenn.edu).

### Materials availability

All mouse lines and reagents generated in this study are available from the lead contact upon completion of a materials transfer agreement.

### Data and code availability

• Source data supporting the findings of this study are accessible and deposited to Mendeley Data, v.1, https://doi.org/10.17632/m8x9yczmf9.1.
• There is no original code in this manuscript.
• Any additional information required to reanalyze the data reported in this paper is available from the lead contact upon request.

## Acknowledgments

The authors would like to thank Caitlin Baumer-Harrison and Samatha Fortin for technical assistance and the various undergraduate research assistants in the Hayes lab for their assistance in husbandry for the animals. This work was supported by the 10.13039/100000062National Institute of Diabetes and Digestive and Kidney Diseases (10.13039/100000062NIDDK), United States 130239 (M.R.H., B.C.D.J., and R.P.D.), T32DK007314 (K.S.S.), and F32DK127591 (C.E.G.).

## Author contributions

Conceptualization was done by K.S.S. and M.R.H. Funding was acquired by K.S.S., B.C.D.J., R.P.D., C.E.G., and M.R.H. Experiments were conducted, and data were acquired and analyzed by K.S.S., O.O., D.B., A.L., G.C., S.K., S.Y., D.V., and T.H.K. Writing of the original draft was done by K.S.S. and M.R.H., and review and editing were done by K.S.S., B.C.D.J., R.P.D., C.E.G., and M.R.H.

## Declaration of interests

M.R.H. and B.C.D.J. receive research funding from Boehinger Ingelheim, Kailera Therapeutics, Eli Lilly & Co., and Protagonist Tx that was not used in support of these studies. M.R.H. and R.P.D. are founding scientists, and B.C.D.J. and C.E.G. are members of the SAB for Coronation Bio, which did not contribute to the financial support of these studies. M.R.H. and R.P.D. are founding scientists of, and B.C.D.J. is a member of the SAM for Melogy Tx (Delaware, USA), which did not contribute to the financial or intellectual support of these studies.

## STAR★Methods

### Key resources table

**Table:** 

REAGENT or RESOURCE	SOURCE	IDENTIFIER
**Biological samples**		

Mouse brain (Rxfp3 fl/fl hsyn-Cre+)	In house (University of Pennsylvania)	N/A
Mouse brain (Rxfp3 fl/fl hsyn-Cre-)	In house (University of Pennsylvania)	CAT# JAX:028543
Mouse brain (hSyn-Cre+)	In house (University of Pennsylvania)	CAT# JAX:003966

**Chemicals, peptides, and recombinant proteins**		

Relaxin-3	Phoenix Pharmaceuticals	CAT# 035–36
Cisplatin	Sigma Aldrich	CAT# 15663–27-1
GDF-15	Abcam	ab302451

**LiCl**		

DMSO	Honeywell	CAT# 34869-1L
Artificial cerebrospinal fluid (aCSF)	Havard Appartus	CAT# 59–7316
Angiotensin II (Ang II)	Bachem	Product #4006473

**Critical commercial assays**		

Fast Advanced Master Mix	Applied Biosystems	CAT# 4444554
iScript	Bio-rad	CAT# 1708891

**Deposited data**		

Raw data for all figures and supplemental figures	Mendeley Data	(https://doi.org/10.17632/m8x9yczmf9.1)

**Experimental models: Organisms/strains**		

Rat (Sprague-Dawley)	Charles River	CAT#: CR:001
Adult male WT mice (C57BL/6)	The Jackson Laboratory	CAT#: JAX:000664
Mouse: RXFP3 fl/fl hSyn-Cre+ mice	Generated in this study	N/A

**Oligonucleotides**		

RXFP3 Taqman probe	Thermofisher	CAT#: Mm00618735_s1
B2M Taqman Probe	Thermofisher	CAT# Mm00437762_m1

**Software and algorithms**		

Biorender	Biorender	https://www.biorender.com/
GraphPad PRISM	GraphPad Software	RRID: SCR_000306
StepOne Software v2.3	Applied Biosystems	RRID:SCR_014281
G∗Power Software	Heinrich-Heine-Universität Düsseldorf	RRID:SCR_013726

**Other**		

Rodent chow	Purina Lab Diet	CAT#: 5001
60% High-Fat/High-Sucrose Diet	Research Diet	CAT#: D12492
Kaolin Clay	Research Diet	CAT# 57–504
BioDAQ	Research Diets	https://www.researchdiets.com/biodaq
Operant Conditioning Boxes	Med Associates	https://www.med-associates.com
45 mg Unflavored, sucrose reward pellets	Bio-Serv	CAT#: F0023

### Experimental model and study participant details

#### Animals

Adult wildtype (∼8 weeks old) maleSprague-Dawley rats (Charles River, RRID:RGD_734476) weighing ∼250–275 upon arrival were individually housed under a 12h-light:12h-dark cycle in a temperature and humidity-controlled satellite vivarium and had *ad libitum* access to water and chow (5001, LabDiet; Calories provided by protein 28.9%, fat 13.6%, carbohydrate 57.5%; 3.35 kcal/g) or a 60% high fat diet (HFD; D12492, Research diets; Calories provided by protein 26.2%, fat 34.9%, carbohydrate 26.3%; 5.24 kcal/g) and when indicated had *ad libitum* access to kaolin pellets (Research Diets, K50001) to assess pica behavior. Rats were exposed to kaolin for at least 5 days prior to testing and only exposed following surgical recovery. Rats that consistently spilled kaolin over 10 g were excluded. Except for studies conducted in the bioDAQ, rats were housed in hanging wire-bottomed cages to allow for accurate measurement of food spillage.

Adult (∼8 weeks old) wildtype male C57BL/6NTac mice (Taconic, RRID:IMSR_TAC:B6), RXFP3 fl/fl (The Jackson Laboratory, RRID:IMSR_JAX:028543), hSyn-Cre+ mice (The Jackson Laboratory, RRID:IMSR_JAX:003966) and RXFP3 fl/fl hSyn-Cre+ mice individually housed under a 12h-light:12h-dark cycle in a temperature and humidity-controlled satellite vivarium and had *ad libitum* access to water and a 60% high fat diet (HFD; D12492, Research diets; Calories provided by protein 26.2%, fat 34.9%, carbohydrate 26.3%; 5.24 kcal/g).

RXFP3 fl/fl hSyn-Cre+ mice were generated by crossing RXFP3 fl/fl and hSyn-Cre+ mice, and offspring from these crosses were genotyped for the presence of the floxxed sequence around the RXFP3 gene and the presence of the hSyn-Cre recombinase. To further verify successful cre-lox recombinase, brains were harvested and central RXFP3 mRNA expression was quantified using qPCR.

All animals used in this study were male. Therefore, the influence of sex or gender on the study outcomes could not be assessed.

All animals were acclimated to single housing conditions and injections at least 1 week prior to experimentation. At all times animals had *ad libitum* water access even when food deprived. Experiments were conducted under the National Institutes for Health Guide for the Care and Use of Laboratory Animals and all procedures were approved by the Institutional Animal Care and Use Committee at the University of Pennsylvania (IACUC Protocol 803503).

### Method details

#### Drugs

Human relaxin-3 was commercially purchased (Cat: 035–36, Phoenix Pharmaceuticals). Relaxin-3 was diluted in artificial cerebrospinal fluid (aCSF, Harvard Apparatus) to 0.1, 1, or 2μg/2 μL for intracerebroventriocular (ICV) injections. For peripheral injections, relaxin-3 was diluted in sterile 0.9% NaCl to 1 or 10 μg/kg. Angiotensin II (Ang II; 4006473, Bachem) was diluted to 10 ng/μL in aCSF. Cisplatin (*Cis*, *cis*-diammineplatinum dichloride, Sigma-Aldrich, 15663–27-1) was dissolved in 0.9% saline (1 mg/2 mL volume) and administered at a dose of 6 mg/kg in rats. These doses of cisplatin were chosen based on previous studies within this range showing reliable induction of behavioral effects.^55^ Central GDF15 (human recombinant, Biovision, cat. 4569) was administered at concentrations of 30 pmol/ul in 100% DMSO (Honeywell, cat. 34869–1L). LiCl (0.15 M in 0.9% saline, 20 mL/kg, Sigma Aldrich, Cat. L4408) was injected intraperitoneally (IP).^56^

#### Surgeries

For all surgeries, rats were anesthetized by intraperitoneal (IP) injection of a mixture containing ketamine (90 mg/kg, Butler Animal Health Supply), xylazine (2.7 mg/kg, Anased), and acepromazine (0.64 mg/kg, Butler Animal Health Supply) (KAX) and then placed into a stereotaxic instrument. Rats were implanted with a unilateral guide cannula (26-ga, Plastics One) targeting the lateral ventricle (guide cannula coordinates: ML: ±1.8 mm, AP: −0.9 mm, and DV: −2.6 mm; internal cannula aimed at −4.6 mm with a 2 mm projection tip). Post-operative surgical recovery consisted of rats receiving post-operative temperature support, subcutaneous saline (5 mL) and analgesia (2 mg/kg meloxicam). Meloxicam was administered daily for 2 days post-surgery and body weight was monitored for 10 days post-surgery. Following post-op recovery, cannula placements were verified with functional *in vivo* testing. Lateral ventricle cannula placement was confirmed by observing polydipsia following Ang II injection. Rats that drank excessively (>5 mL) within 10 min of a lateral ventricle Ang II injection (10 ng/μL) passed while any rats that failed to do so were excluded from further study.

#### RT-QPCR

RT-qPCR was performed using TaqMan Fast Advanced Master Mix, no UNG (Thermo Fisher Scientific, Catalog number A44360) on Applied Biosystems StepOnePlus Real-Time PCR System and StepOne Software v2.3. Gene expression was normalized to the indicated housekeeping gene using the ΔΔCT method. Primer sequences were purchased commercially (Thermo Fisher Scientific, Mm00618735_s1, Catalog number 4331182). Tissue brain punches of the hindbrain, hypothalamus and ventral hippocampus were taken from WT-Syn-Cre+, RXFP3 fl/fl Syn-Cre- and RXFP3 fl/fl Syn-Cre+ mice. The relative amount of transcript levels was calculated using the delta/delta CT method and normalized to B2M levels.

#### General *in vivo* design

Animals were habituated to single housing in their home cage and received mock (no volume injected) intraperitoneal (IP), subcutaneous (SC) or intracerebroventricular (ICV) lateral injections at least 1 week prior to experimentation. All behavioral experiments used a counterbalanced, within-subjects, with each treatment separated by 72 h. For all feeding experiment days, animals were food deprived 2 h before the dark cycle onset and injections were done immediately prior to the dark cycle onset. For all feeding experiments body weight was measured immediately before and 24 h after injection. For feeding experiments excluding BioDAQ studies, food and/or kaolin intake was measured at various time points between 1–24 h after injections were completed and for studies with rats, food crumbs were weighed and accounted for at each timepoint. For BioDAQ studies, food intake was automatically recorded and water intake (bottle weight) was measured immediately before and 24 h after injection.

#### Acute lateral ventricle and systemic injections of relaxin-3 on high-fat diet food intake in rats

Rats were housed in the BioDAQ. The BioDAQ system records on a second-by-second basis for undisturbed measurements of episodic food intake. Individual bouts are initiated by the animal at onset and termination of feeding; bouts are separated by 5 s inter-bout interval (IBI). A meal is defined as at least one bout with a minimum meal size of 0.2g and separated by a 5-min undisturbed inter-meal interval (IMI). Cumulative food intake was calculated at 30min, 1h, 1.5h, 2h, 2.5h, 3h, 4h, 5h, 6h and 24-hs following injections. Additionally, the number of meals, time spent consuming meals (sec), average meal size (g/meal), and average meal length (sec/meal) were calculated for the following time intervals (0-30min,0-1h, 0-1.5h,0-2h, 0-2.5h, 0-3h, 0-4h, 0-5h,0-6h and 0–24 hs) relative to drug administration. The latency to consume the first meal and bout and the size of the first meal and bout in the dark cycle were also calculated. Intracerebroventricular (ICV) lateral or subcutaneous (SC) injections of either relaxin-3 or vehicle (ICV; aCSF and SC: 0.9% saline) were done immediately prior to the dark cycle onset. Intracerebroventricular (ICV) injections were administered manually using a Hamilton syringe terminating in an injector tip extending 2.0 mm beyond the guide cannula. Body weight was measured immediately before and 24 h after injections.

#### Acute systemic injections of relaxin-3 in RXFP3 fl/fl hSyn-Cre+ mice

Using a counterbalanced, within-subjected design, RXFP3 fl/fl Syn-Cre+ mice and diet-induced obese (DIO) wildtype mice were intraperitoneally (IP) injected with either relaxin-3 or vehicle (0.9% saline). Food was measured at 1,3,6 and 24hs after injections and body weights were recorded at the 24hs timepoint. Food was removed 2h prior to lights going off and baseline food and body weights were recorded.

#### Effects of lateral ventricle injections of relaxin-3 on cisplatin-induced anorexia and body weight loss in rats

To evaluate the effects of central relaxin-3 to combat systemic cisplatin-induced cachexia, rats maintained on standard chow were pretreated with ICV lateral injections of vehicle (aCSF) or relaxin-3 30min prior to IP injections of saline (*n* = 19). Since systemic injections of cisplatin is a terminal procedure, rats were then split using a counterbalanced, pseudo-between subjects design to be pretreated with ICV lateral injections of vehicle or relaxin-3 30min prior to IP injections of cisplatin (n = 6–7). Food and kaolin intake was recorded at 3h, 6h and 24hs post injections. Body weight was recorded 24h post injection. Animals were euthanized 2 days post cisplatin injection.

#### Progressive ratio

The PR protocol was modified from previously described work.^57^ The training sessions were conducted in operant conditioning boxes (Med Associates; Fairfax, VT, USA). Each training session was 1 h and there was a total of 6 training sessions. The first two training sessions were on an FR1 schedule with autoshaping, where each lever press was reinforced with a 45-mg pellet (35% kcal from fat enriched with sucrose, F05989, Bio-Serv, Frenchtown, NJ) and the autoshaping consisted of a 45-mg pellet being dispensed after 600s without a lever-press. The third and fourth training sessions were on an FR1 schedule without autoshaping. The fifth and sixth training sessions were on an FR3 schedule, where 3 presses were required for each pellet earned. During the test session, the response requirement of the PR schedule increased progressively using the following formula: *F*(*i*) = 5eˆ0.2*i*–5, where *F*(*i*) is the number of lever presses required for next pellet at *i* = pellet number. The breakpoint for each animal was defined as the highest F(i) completed that preceded a 20-min period without earning a reinforcer. For all procedures, there was an active (reinforced) and an inactive (non-reinforced) lever.

On test day, food was removed 2 h prior to the lights going off and behavioral testing began at lights offset. Animals were randomized to receive either aCSF or relaxin-3 using a counterbalanced within-subjects design, with a washout period of 72 h between treatments 1 and 2. Injections were given 45 min prior to behavioral testing, and food was returned after the testing was complete.

#### Binge-eating paradigm

Rats were counterbalanced based on body weight and food intake and split into two groups: a control daily group and a binge group. The control daily group received 1h access to Crisco every day and the binge group received 1h of Crisco every other day. Chow and Crisco intake were measured for six weeks. After six weeks, within each group, animals received either ICV lateral aCSF or relaxin-3 using a counterbalanced within-subjects design. Crisco was measured 1h after injections, and Chow was measured 1, 3, 6 and 24h after injections with body weight also being recorded at 24h after injections.

### Quantification and statistical analysis

All data are reported as means ± SEM and statistical analyses for the data presented in Figures 1, 2, 3, 4, 5 and S1–S3 were done with the assumption that the data was normally distributed and had equal standard deviations. Statistical significance was determined using unpaired or paired 2-tailed Student’s t tests for comparison between two groups. For comparison between more than two groups, either one-way or two-way ANOVAs with either Tukey’s, Dunnett’s or SNK *post hoc* analysis was used (GraphPad Prism Software Inc.). All sample size (n), and specific statistical tests used can be found in the figure legends of the corresponding figure. Power calculation analysis was determined using G∗Power open-source software program based on pilot data and the literature on the feeding effects of relaxin-3. For all analyses, a *p*-value <0.05 was considered statistically significant (∗*p* < 0.05, ∗∗*p* < 0.01, ∗∗∗*p* < 0.001, ∗∗∗∗*p* < 0.0001).
