# Supplementary material for: Central relaxin-3/RXFP3 signaling potently drives orexigenic behaviors and attenuates cisplatin-induced anorexia and body weight loss
Source: iScience. 2026 Oct 8;29(11):117785. doi: 10.1016/j.isci.2026.117785 (PMC13676733; doi:10.1016/j.isci.2026.117785)
Supplement: Supplementary file 1 [file mmc1.pdf]

iScience, Volume *29*

Supplemental information

Central relaxin-3/RXFP3 signaling potently drives orexigenic behaviors and attenuates cisplatin-induced anorexia and body weight loss

Keshav S. Subramanian, Drew Belser, Oleksandr Orativskyi, Ashley Lee, Genevieve Curtis, Schuylar Kamicha, Shion Yuki, Dennis Vu, Tsz Hang Ko, Bart C. De Jonghe, Robert P. Doyle, Caroline E. Geisler, and Matthew R. Hayes

Supplemental 1

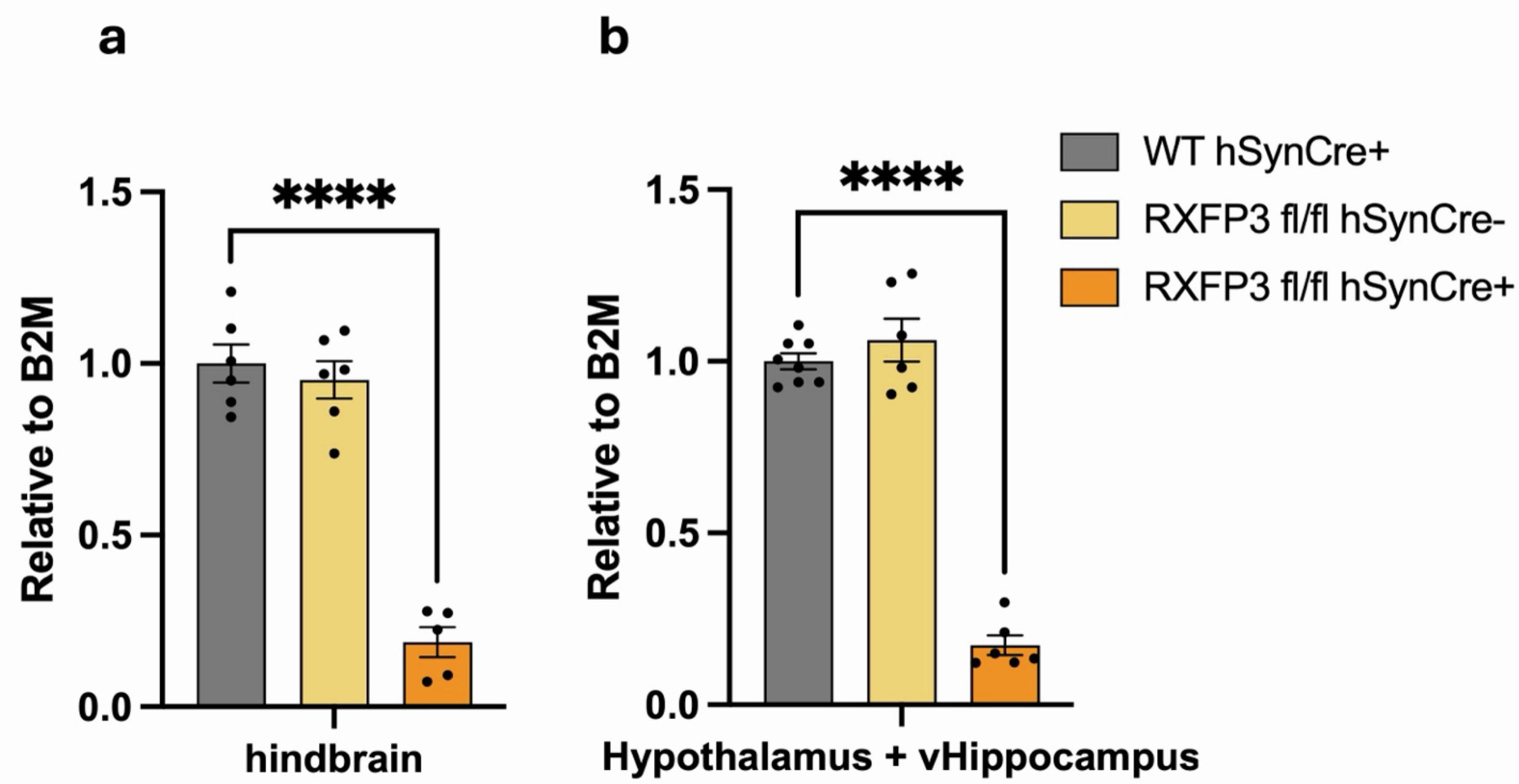

Supplemental 1: Verification of specific neuronal RXFP3 deletion (Related to Figure 3)
Neuronal RXFP3 expression is deleted in RXFP3 fl/fl Syn-Cre+ mice. Relative to B2M (housekeeping gene), RXFP3 mRNA expression in the (a)hindbrain and (b) hypothalamus and ventral hippocampus in WT hSyn-Cre+, RXFP3 fl/fl hSyn-Cre- and RXFP3 fl/fl hSyn-Cre+ mice (n=6-8). Data are means ± SEM. Data were analyzed by one way ANOVA with Dunnett's post-hoc (**** $P < 0.0001$).

Supplemental 2

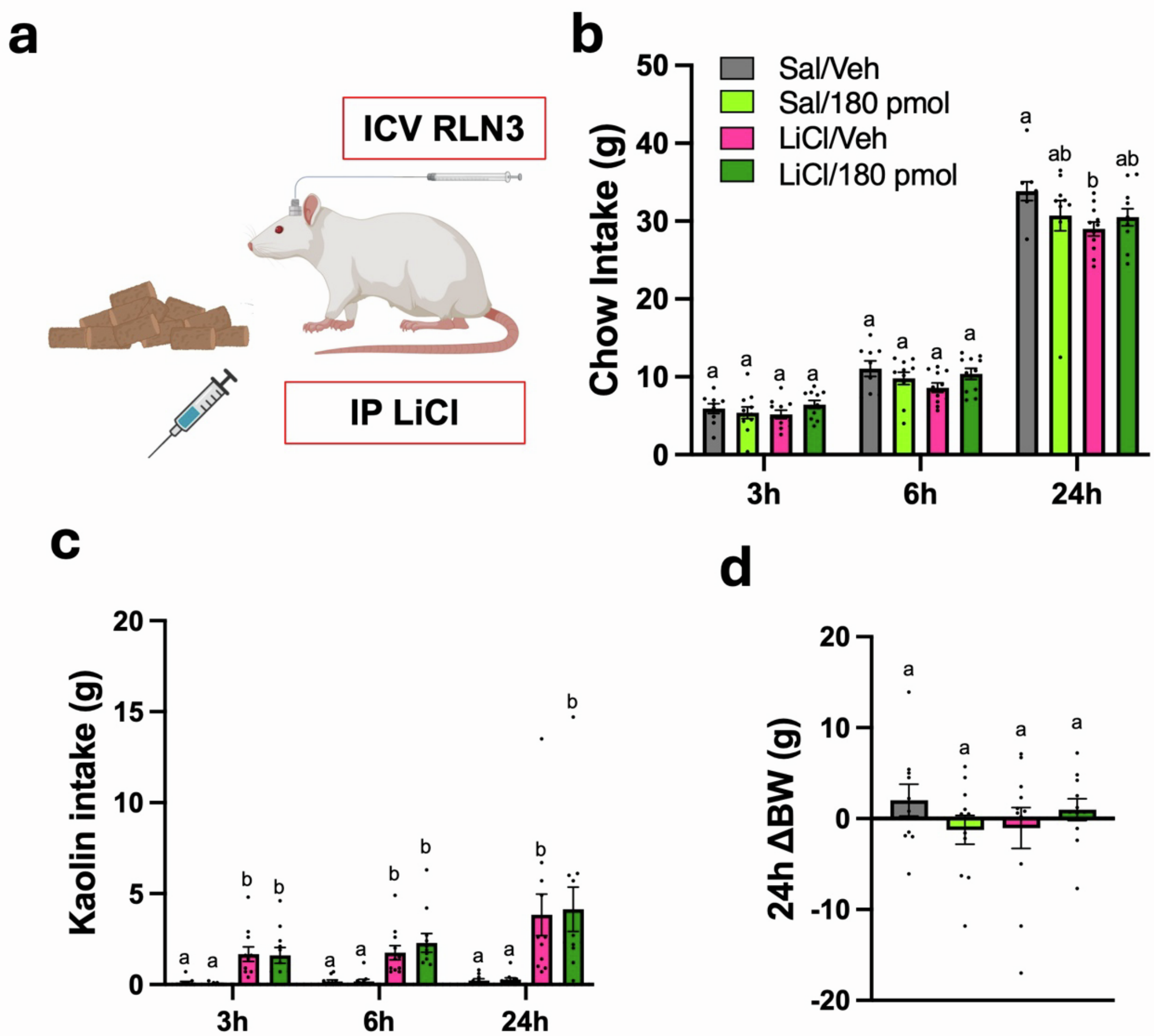

Supplemental 2: Central relaxin-3 does not attenuate LiCl-induced anorexia and nausea (Related to Figure 5)

Central relaxin-3 (180pmol/2µL) administration does not impact LiCl (20ml/kg) induced anorexia and body weight loss. (A) Schematic diagram depicting pretreatment of ICV lateral administration of relaxin-3 with systemic intraperitoneal administration of LiCl in rats maintained on standard chow (n=10per group). (B) chow intake (C) laolin intake and (D) 24-hour body weight change. Data are means ± SEM. Data were analyzed by two-way [(B), (C)] and one-way ANOVA with Tukey or Dunnett's post hoc. Bars that do not share a common letter (a, b, and c) within a time point differ significantly ($P \leq 0.05$). Sal, saline; Veh, vehicle; LiCl, lithum chloride; 1µg = relaxin-3 (1µg/2µL); h, hour.

Supplemental 3

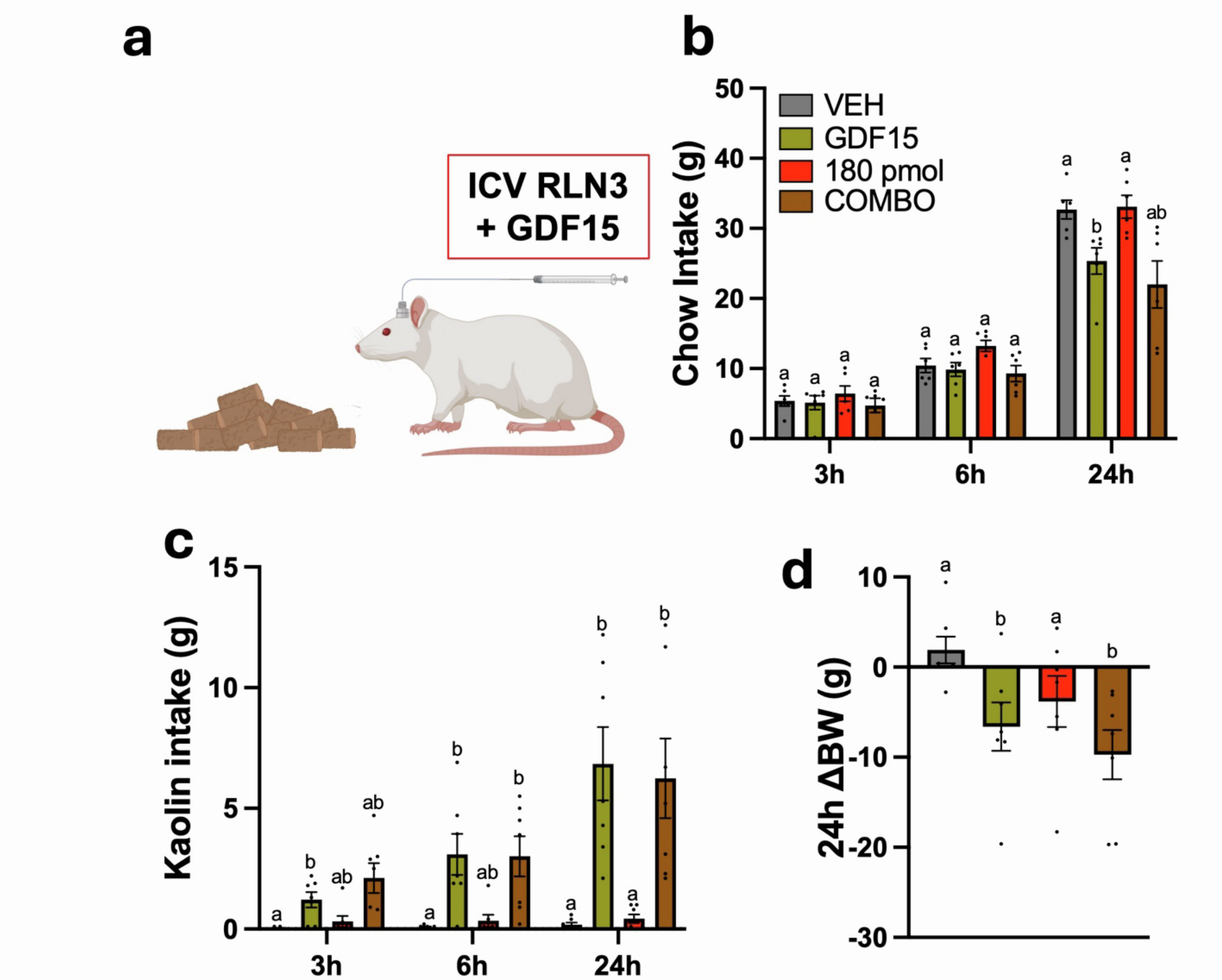

Supplemental 3: Central relaxin-3 does not attenuate GDF-15-induced anorexia, nausea and body weight loss (Related to Figure 5)

Central relaxin-3 (1µg/2µL) administration does not impact GDF (30pmoll/2µL) induced anorexia and body weight loss. (A) Schematic diagram depicting pretreatment of ICV lateral administration of relaxin-3 with ICV lateral administration of GDF15 in rats maintained on standard chow (n=6 per group) (B)Chow intake (C) Kaolin intake and (D) 24-hour Body weight change. Data are means ± SEM. Data were analyzed by two way [(B) to (D)l ANOVA with Tukey post hoc.
